# An exploratory study of volumetric analysis for assessing tumor response with ^18^F-FAZA PET/CT in patients with advanced non-small-cell lung cancer (NSCLC)

**DOI:** 10.1186/s13550-016-0187-6

**Published:** 2016-04-18

**Authors:** Gerald S. M. A. Kerner, Vikram R. Bollineni, Thijo J. N. Hiltermann, Nanna M. Sijtsema, Alexander Fischer, Alphons H. H. Bongaerts, Jan Pruim, Harry J. M. Groen

**Affiliations:** Department of Pulmonary Diseases, University Medical Center Groningen, University of Groningen, Hanzeplein 1, P.O.Box 30.001, 9700 RB Groningen, The Netherlands; Department of Radiation Oncology, University Medical Center Groningen, University of Groningen, Groningen, The Netherlands; Philips Technologie GmbH Innovative Technologies, Aachen, Germany; Department of Nuclear Medicine and Molecular Imaging, University Medical Center Groningen, University of Groningen, Groningen, The Netherlands; Department of Nuclear Medicine, Tygerberg Hospital, Stellenbosch University, Stellenbosch, South Africa

**Keywords:** ^18^F-FAZA, ^18^F-FDG, NSCLC, Chemotherapy

## Abstract

**Background:**

Hypoxia is associated with resistance to chemotherapy and radiotherapy and is randomly distributed within malignancies. Characterization of changes in intratumoral hypoxic regions is possible with specially developed PET tracers such as ^18^F-fluoroazomycin arabinoside (^18^F-FAZA) while tumor metabolism can be measured with 2-deoxy-2-[^18^F]fluoro-d-glucose (^18^F-FDG). The purpose of this study was to study the effects of chemotherapy on ^18^F-FAZA and ^18^F-FDG uptake simultaneously in non-small-cell lung cancer (NSCLC) patients

**Methods:**

At baseline and after the second chemotherapy cycle, both PET/CT with ^18^F-FDG and ^18^F-FAZA was performed in seven patients with metastasized NSCLC. ^18^F-FAZA and ^18^F-FDG scans were aligned with deformable image registration using Mirada DBx. The primary tumors were contoured, and on the ^18^F-FDG scan, volumes of interest (VOI) were drawn using a 41 % adaptive threshold technique. Subsequently, the resulting VOI was transferred to the ^18^F-FAZA scan. ^18^F-FAZA maximum tumor-to-background (T/Bg_max_) ratio and the fractional hypoxic volume (FHV) were assessed. Measurements were corrected for partial volume effects. Finally, a voxel-by-voxel analysis of the primary tumor was performed to assess regional uptake differences.

**Results:**

In the primary tumor of all seven patients, median ^18^F-FDG standard uptake value (SUV_max_) decreased significantly (*p* = 0.03). There was no significant decrease in ^18^F-FAZA uptake as measured with T/Bg_max_ (*p* = 0.24) or the FHV (*p* = 0.35). Additionally, volumetric voxel-by-voxel analysis showed that low hypoxic tumors did not significantly change in hypoxic status between baseline and two cycles of chemotherapy, whereas highly hypoxic tumors did. Individualized volumetric voxel-by-voxel analysis revealed that hypoxia and metabolism were not associated before and after 2 cycles of chemotherapy.

**Conclusions:**

Tumor hypoxia and metabolism are independent dynamic events as measured by ^18^F-FAZA PET and ^18^F-FDG PET, both prior to and after treatment with chemotherapy in NSCLC patients.

## Background

Hypoxia is considered an important feature of malignant tumors with direct influence on the efficacy of chemo- and radiotherapy. Hypoxia is unevenly distributed within tumors, that can be related to an abnormal vasculature and to an elevation in interstitial pressure associated with tumor growth [1]. Hypoxic tumor cells are associated with more aggressive phenotypes and with resistance to both chemo- and radiotherapy [2].

The gold standard in assessing tumor hypoxia is the polarographic Eppendorf electrode method. Unfortunately, this technique is rather invasive, and therefore, it can only be applied in well-accessible superficial tumors. It measures oxygen concentrations at the site of the electrode only and therefore is not indicative for the whole tumor. Consequently, multiple punctures are necessary to measure heterogeneity in oxygen distribution within a tumor. These shortcomings may be overcome by using a non-invasive technique such as PET radiotracers aimed at visualizing and quantifying hypoxia. Different radiotracers have been developed, most of them based on nitroimidazole compounds. The intracellular retention of these compounds is based on the oxygen concentration with tissue retention (in vitro) in hypoxic conditions observed up to 28 times normal uptake [3, 4]. Nitroimidazole tracers are diffused through the cell membranes and then undergo reduction to yield radical anions. Under normoxic conditions, these radical anions are reoxidized and diffuse out of the cell. However, in a hypoxic environment, re-oxidation cannot occur and the radicals are irreversibly bound to macromolecules, resulting in increasing tracer accumulation under decreasing oxygen concentration. The most widely used PET hypoxia tracer from this group is ^18^F-fluoromisonidazole (^18^F-FMISO). Another tracer is ^18^F-fluoroazomycin arabinoside (^18^F-FAZA) [5–9], which compared to ^18^F-FMISO has a more favorable signal-to-noise ratio [6, 10].

Hypoxia and glucose metabolism are distinct metabolic processes, yet it seems closely interlinked due to the so called Warburg effect [11]. The Warburg effect comes from the observation that cancer cells produce energy using anaerobic glycolysis, instead of oxidative phosphorylation at the cost of increased glucose uptake. The link between hypoxia and glucose metabolism is through the hypoxia inducible factor (HIF). The HIF complex activates the transcription of multiple genes that are necessary for the Warburg effect to occur [12]. Consistent HIF pathway activation in tumors is related to the expression of two different receptors viz., GLUT-1 and GLUT-3 as well as regulation of enzymes in the glycolytic pathway [13–15]. There is a clear relationship between 2-deoxy-2-[^18^F]fluoro-d-glucose (^18^F-FDG) uptake and especially GLUT-1 and also with GLUT-3 expression [16]. This suggests an interdependency of both processes, and consequently, ^18^F-FDG uptake could function as a biomarker of hypoxia. Indeed, such a relationship has been shown [17]. However, a number of recent publications failed to show a clear relation between the two tracers [8, 18–20].

Traditionally, in most patient studies, measurement of the maximum and/or mean standard uptake value (SUV) or the tumor-to-background (T/Bg) mean ratio is used. Despite their widespread use, they all have a drawback in the sense that heterogeneity of tumor metabolism is ignored. Alternatively, assessing localized treatment effects in the individual tumor areas may be possible by using volumetric voxel-by-voxel tumor analysis techniques.

Previously, our group studied the relationship between ^18^F-FAZA and ^18^F-FDG uptake in untreated patients using volumetric analysis [18]. Chemotherapy is standard treatment in advanced non-small-cell lung cancer (NSCLC), and the impact on hypoxia and metabolism within individual tumor areas has not been adequately addressed.

The purpose of this paper is therefore to study the relation between hypoxia and metabolism by a voxel-by-voxel approach in a clinical setting with ^18^F-FDG and ^18^F-FAZA. We compared this analysis with the traditionally measured SUV_max_ and T/Bg. As a model for this comparison, we chose to use patients with advanced NSCLC.

## Methods

### Patients

Treatment-naive patients with advanced stage IV NSCLC were selected. All patients underwent PET scans with both ^18^F-FAZA and ^18^F-FDG at baseline and after 2 cycles of chemotherapy. The study was approved by the Institutional Review Board of the University Medical Center Groningen. All patients gave written informed consent prior to study participation.

### ^18^F-FDG PET/CT

^18^F-FDG PET was performed on a Siemens Biograph mCT 64. ^18^F-FDG images were reconstructed according to EANM guidelines [21]. Blood samples were taken before tracer injection to confirm acceptable blood glucose level (<11 mmol/l) after a minimal 4 h fasting period. Patients were injected with 3 MBq/kg bodyweight ^18^F-FDG intravenously. After 60 min, a scan was made from the mid-thigh to the brain. Scan times per bed position were dependent on patient weight: 1 min per bed position if less than 60 kg, 2 min if between 60 and 90 kg, and 3 min if above 90 kg [22]. PET data were reconstructed as described previously [18].

SUVs were obtained by delineating the tumor volume of interest (VOI) comprising the entire tumor volume using the IMALYTICS Research Workstation (Philips Technologie GmbH Innovative Technologie, Aachen, Germany) on ^18^F-FDG PET/CT images. The primary tumor area was selected and the VOI delineated using the 41 % adaptive threshold technique as described by Cheebsumon et al [23]. SUVs were normalized for blood glucose levels in accordance with EANM guidelines [21]. If the tumor size in the shortest axis was smaller than 30 mm, the SUV_max_ was corrected for partial volume based on historical data obtained with a NEMA phantom [18]. PET tumor response was assessed according to the 1999 EORTC recommendations [24]. The volume for determination of the background activity was delineated posterior of the sternum in the mediastinal fat tissue using the low-dose CT. This volume was chosen in order to avoid possible confounding by active tumor within the lymph node, and confounding due to artherosclerotic plaques within the great mediastinal vessels. This background volume had to be at least 14 ml.

Contrast-enhanced CT was performed consecutively in the same session on the Somatom CT which is part of the mCT. Scan time was 8 s, craniocaudally at inspiration. Effective mAs was 80 and 120 kV with the care dose setting was used. Slice thickness was 0.5 mm, and pitch was 1.4 with a rotation of 0.5 s. Patients were injected with 55 ml of Iomeron contrast 350 mg/ml (Bracco Imaging Deutschland GmbH, Konstanz, Germany) at a speed of 2.5 ml/s. The tumor size of the primary tumor and response was measured according to RECIST 1.1 criteria [25].

The baseline scan was performed before any therapy was given as part of the diagnostic workup. The scan after 2 cycles of chemotherapy was performed within 2 days after the second cycle of chemotherapy.

### ^18^F-FAZA PET/CT

^18^F-FAZA PET scans were made on the same mCT machine as described previously [18]. Patients received 370 MBq ^18^F-FAZA intravenously. After 120 min, a scan was made from the mid-thigh to the brain. The same ^18^F-FDG scan patient position and fixation system were used in order to improve reproducibility. The baseline scan was performed as soon as possible after inclusion (median 2 days after ^18^F-FDG PET). The scan after 2 cycles of chemotherapy was performed within 3 days after the second cycle of chemotherapy to study the influence of chemotherapy on the FAZA distribution in the primary tumor (median 1 day after ^18^F-FDG PET).

### Deformable image registration

Deformable image registration was performed using Mirada DBx Build 1.1.1.3 (64 bit) (Mirada Medical Ltd, Oxford, UK). First, an automatic rigid registration was performed, followed by a CT-based deformable registration. For each patient, the ^18^F-FDG and ^18^F-FAZA scans obtained at baseline and after 2 cycles of chemotherapy were registered and deformed. This resulted in four deformed images:

1. Between ^18^F-FDG and ^18^F-FAZA at baseline
2. Between ^18^F-FDG and ^18^F-FAZA after 2 cycles of chemotherapy
3. Between ^18^F-FDG at baseline and after 2 cycles of chemotherapy
4. Between the deformed ^18^F-FAZA at baseline generated from step 1 and the unaltered ^18^F-FAZA images after 2 cycles of chemotherapy

The registered and deformed images were then imported back into the Imalytics system for further analysis.

### Voxel-by-voxel analysis

After the registration and deformation of the images, each voxel in a VOI of the ^18^F-FAZA images was normalized to the mean background value (obtained from corresponding VOIs posterior of the sternum) in order to calculate a T/Bg. This enables a regional comparison between ^18^F-FDG SUV (normalized to glucose) and ^18^F-FAZA T/Bg. Partial volume correction was applied to the T/Bg data using the same method as for the ^18^F-FDG PET data.

For regional comparison between baseline and follow-up scans, the tumor VOI delineated in the baseline ^18^F-FDG was transferred to the ^18^F-FAZA after image registration as described previously. To guarantee the use of operator-independent reproducible VOI, the tumor VOI delineated on the baseline ^18^F-FDG images using an automated method was used on the baseline to follow up scan comparison of ^18^F-FAZA. To assess the regional comparison between the four different deformed images with their respective reference images, the voxel-by-voxel comparison capabilities of the propagated align algorithm of the IMALYTICS Research Workstation was used. The function of the propagated align algorithm was previously described by our group [26].

The T/Bg_max_ as well as the fractional hypoxic tumor volume (FHV) was calculated using the voxel-by-voxel analysis data (^18^F-FDG to ^18^F-FAZA, if this data was not available then ^18^F-FAZA to ^18^F-FAZA). The ^18^F-FAZA T/Bg_max_ was corrected for PVE using the same technique as for the ^18^F-FDG SUV_max_ data. The FHV was defined as the fraction of the tumor volume exceeding a T/Bg ratio of 1.4. In previous studies by our group, the cutoff of 1.4 was used to define hypoxia [18, 27].

### Statistics

Pre- and post-chemotherapy ^18^F-FDG and ^18^F-FAZA scans using the maximum uptake values were analyzed with the Wilcoxon signed rank test. The voxel-by-voxel relationship per patient between the ^18^F-FDG SUV and the ^18^F-FAZA T/Bg at baseline and after 2 cycles of chemotherapy was calculated using simple linear regression. All calculations were performed using SPSS 22.0 (International Business Machines Corp, Armonk, NY, USA). *p* values <0.05 were considered statistically significant.

## Results

### Patient characteristics

Seven patients with advanced stage IV NSCLC were included. Six patients had adenocarcinoma, and one had large cell lung carcinoma. Patient details are given in Table 1. Chemotherapy consisted of a platinum containing doublets. Partial response according to RECIST was observed in one patient, stable disease in four patients, and progressive disease in two patients.

**Table 1:** Comparison of the effect of chemotherapy on the primary tumor measured with ^18^F-FAZA and ^18^F-FDG PET including tumor response in non-small-cell lung cancer

Patient	Age	Sex	^18^F-FAZA (T/Bg ratio)^a^		^18^F-FDG (SUV_max_)^b^		Fractional hypoxic volume		CT scan
			Baseline	2 cycles	Baseline	2 cycles	Baseline	2 cycles	
1	56	F	7.1	5.0	32.5	24.2	96 %	100 %	PD
2	43	M	3.0	2.0	15.4	2.1	98 %	9 %	SD
3	45	F	4.4	3.3	27.3	18.2	95 %	95 %	PD
4	52	F	7.7	7.7	5.8	^c^	100 %	97 %	SD
5	48	F	2.9	2.6	7.2	5.9	63 %	30 %	SD
6	61	M	6.7	4.7	15.8	4.4	100 %	100 %	SD
7	62	F	3.3	5.4	8.9	6.0	58 %	100 %	PR

*PR* partial response, *SD* stabile disease, *PD* progressive disease

^a^Uptake for the whole primary tumor was corrected for partial volume effect. It was then divided by the mean of the background activity and this is tumor to background (T/Bg) value

^b^Uptake for the whole primary tumor, expressed in SUV, were corrected for partial volume effect

^c^Due to a dosimeter error, not usable for qualitative comparison

### Pre- and post-chemotherapy differences in ^18^F-FAZA and ^18^F-FDG uptake using traditional (max and mean uptake) methods

^18^F-FDG uptake (SUV_max_) between baseline and after 2 cycles of chemotherapy decreased significantly (*p* = 0.03) (Table 1). No differences in T/Bg_max_ (*p* = 0.24) or FHV (*p* = 0.35) were observed when comparing ^18^F-FAZA uptake at baseline and after 2 cycles of chemotherapy.

### Pre- and post-chemotherapy uptake analyzed voxel-by-voxel

**Fig. 1:**
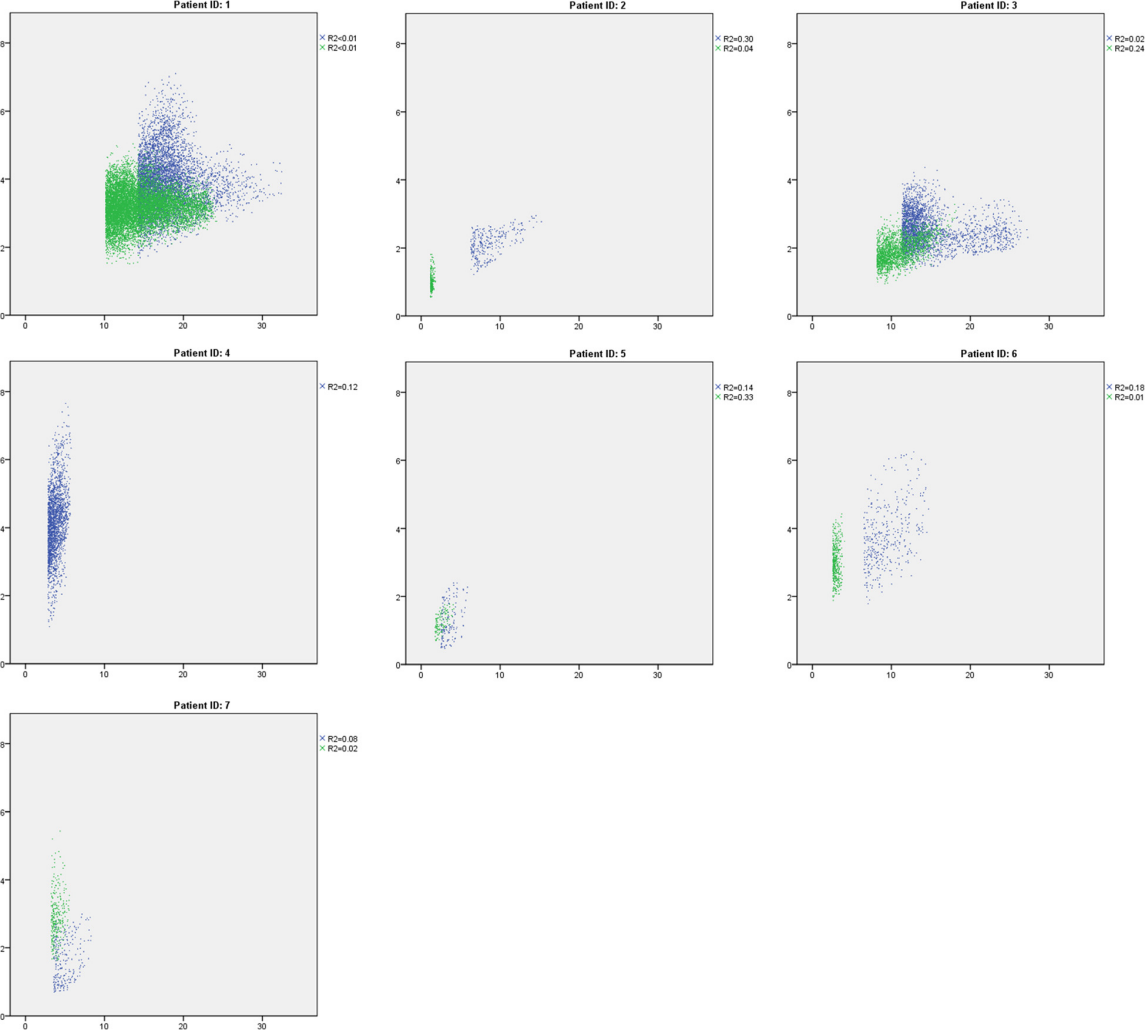
Per patient comparison of regional voxel distribution in the primary tumor of ^18^F-FAZA (T/Bg, vertical axis) versus ^18^F-FDG (SUV, horizontal axis) at baseline and after 2 cycles of chemotherapy. The horizontal axis represents the ^18^F-FDG uptake in SUV. The vertical axis represents the ^18^F-FAZA uptake in tumor-to-background ratio. *Blue* represents voxel distribution assessed at baseline, while *green* represents voxel distribution assessed after 2 cycles of chemotherapy. ^18^F-FDG data of patient 4 was not available after 2 cycles of chemotherapy

In pre-chemotherapy in 4/7 patients, a weak relationship between FAZA T/Bg and ^18^F-FDG SUV was observed with *R*^2^ values between 0.12 and 0.30 (Fig. 1). After 2 cycles chemotherapy, only in two patients, *R*^2^ was 0.24 and 0.33; in the other patients, no relationship was observed between ^18^F-FDG SUV and ^18^F-FAZA T/Bg (Fig. 1). In one patient, a very weak relationship between ^18^F-FAZA T/Bg and ^18^F-FDG SUV persisted at baseline and after 2 cycles of chemotherapy (*R*^2^ = 0.14 and *R*^2^ = 0.33, respectively).

**Fig. 2:**
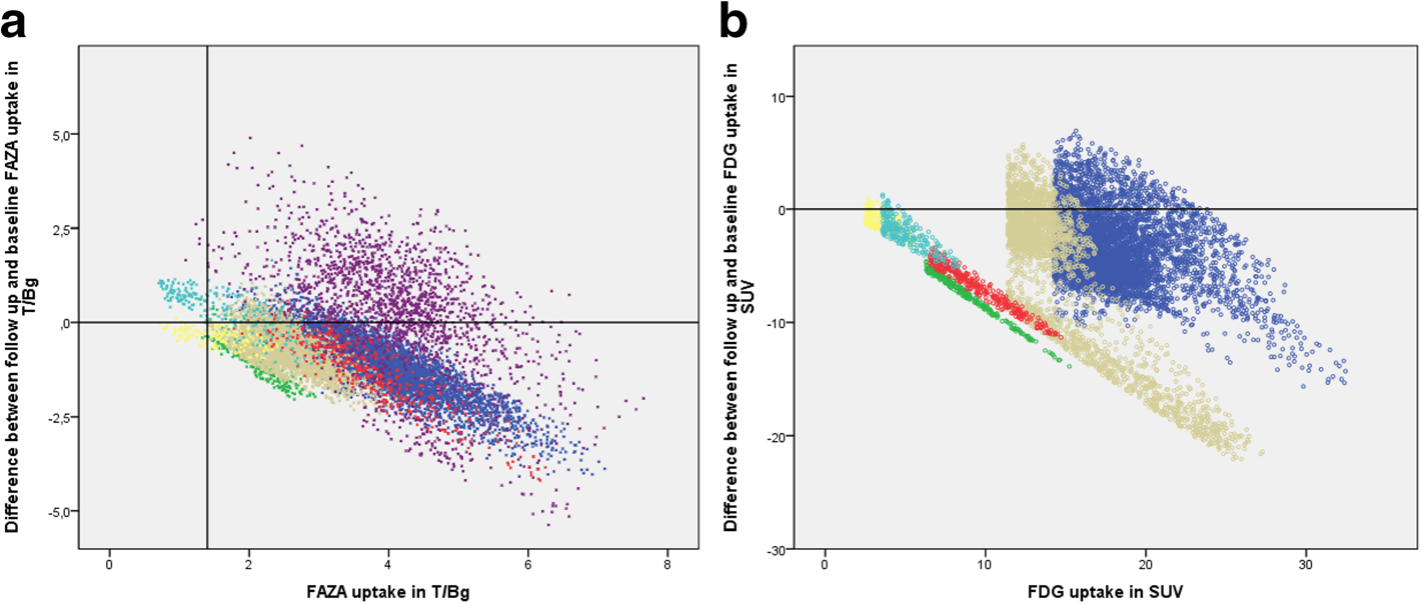
Difference between two cycles of chemotherapy and baseline was assessed for ^18^F-FAZA T/Bg (**a**) and ^18^F-FDG SUV (**b**) in the all primary tumor voxels of seven (^18^F-FAZA) and six (^18^F-FDG) patients, respectively. **a** The *vertical line* in figure (**a**) denotes the hypoxic cutoff of 1.4 T/Bg. Areas above 1.4 T/Bg are considered hypoxic. A decrease in ^18^F-FAZA activity can be seen as a result of treatment, yet this is not so clear compared to ^18^F-FDG. The decrease in ^18^F-FAZA activity is, however, most pronounced in the most hypoxic areas. **b** Per patient, different cutoff values are seen, due to the use of the 41 % adoptive threshold technique in selecting tumor areas. A clear decrease in ^18^F-FDG activity in almost all tumor voxels as a result of treatment. Although the decrease is clear overall, the decrease is most pronounced in areas with initially the highest ^18^F-FDG uptake

In areas with low ^18^F-FAZA uptake at baseline, a decrease of ^18^F-FAZA responses were seen, perhaps partly due to noise. In areas with high ^18^F-FAZA uptake, reoxygenation became evident after chemotherapy (Fig. 2a). A more evenly distributed decrease of ^18^F-FDG activity was observed after 2 cycles of chemotherapy (Fig. 2b). Four different patterns of hypoxic uptake were visible: hypoxic areas that remained hypoxic, hypoxic areas that became normoxic after treatment, normoxic areas that became hypoxic after treatment, and normoxic areas that remained normoxic (Table 2).

**Table 2:** Per patient percentage change between baseline and after 2 cycles of chemotherapy in tumor hypoxic status

Pt. ID.	Normoxic area that remained normoxic (%)	Normoxic area that became hypoxic (%)	Hypoxic area that became normoxic (%)	Hypoxic area that remained hypoxic (%)
1.	0	0	0	100
2.	<1	1	84	15
3.	0	0	18	82
4.	0	<1	3	97
5.	37	0	33	30
6.	0	0	<1	100
7.	1	41	2	56

Hypoxic/normoxic cutoff was defined as 1.4 T/Bg ratio

## Discussion

In this proof-of-concept study, we analyzed whether hypoxia is associated with metabolism by the use of a voxel-by-voxel technique and traditional maximum intensity SUV and T/Bg calculations. Our results show that the technique can be used in a clinical setting. Heterogeneity in ^18^F-FDG distribution could easily be assessed and the effects of chemotherapy varied with the metabolic status of the tumor.

Our study is novel because it is to our knowledge the first study that used ^18^F-FAZA volumetric tumor analysis besides the traditional maximum intensity methods to study the effects of chemotherapy alone on hypoxia in NSCLC patients.

### Hypoxia and metabolism: chemotherapeutic effects

The image analyses showed a very dynamic tumor hypoxia and metabolism illustrating the tracer uptake heterogeneity. Voxel-by-voxel analysis showed no association or at best a very weak association between the two tracers (*R*^2^ ≤ 0.33). After chemotherapy, the metabolic activity decreased within tumor areas, but the hypoxic reactions remained very dynamic. The distribution of hypoxic areas changed in the primary tumor independently of metabolism. Examination by volumetric voxel-by-voxel analysis revealed that tumor regions with initial high ^18^F-FAZA uptake tended to have a larger decrease in the^18^F-FAZA uptake than regions with an initial low ^18^F-FAZA uptake, whereas a random distribution around the overall baseline activity was observed. With traditional analytic methods, hypoxia was also not associated with metabolism [8, 18–20].

There are three factors explaining the lack of a relationship. Most importantly, the Warburg effect occurs even in the abundant presence of oxygen [12]; thus, increased ^18^F-FDG uptake indicative of the Warburg effect does not necessarily reflect hypoxia. Secondly, ^18^F-FDG uptake is not only related to tumor cell uptake but also related to other characteristics which are involved with poor prognosis. Busk et al. investigated with different tumor models in mice the correlation between hypoxia using pimonidazole staining and ^18^F-FDG uptake and found a correlation with an *r* of 0.15 [28]. These uptake characteristics include immune cells/inflammation, local cell density, high relative glycolytic rates, and extracellular acidosis [29, 30]. Importantly, ^18^F-FDG is a nonspecific tracer yet identifies complimentary factors related to prognosis. The third item is that nitroimidazole compounds cannot differentiate between chronic and acute hypoxia. Acute (transient) hypoxia and chronic hypoxia can both co-exist within the same tumor [31]. An in vivo study by Lin et al. demonstrated that acute or chronic hypoxia had differential effects on the distinct HIF proteins (HIF-1α and HIF-2α) [32]. In addition, tumors have areas of hypoxia/anoxia and normoxia, yet part of the tumor is within variable intermediate oxygen levels [33]. The different effects of acute and chronic hypoxia on HIF proteins and subsequently on GLUT-1 expression may provide an explanation why hypoxia and metabolism, while closely interlinked, are not necessarily simultaneously measurable. This explains why ^18^F-FDG alone is not capable of discriminating between hypoxic and non-hypoxic regions in NSCLC and stresses the need for a specific hypoxia identifying imaging agent.

### Volumetric analysis

This study indicates that volumetric analysis may bring more understanding of tumor biology than the other routine techniques will do. Using co-registered and deformed ^18^F-FAZA besides ^18^F-FDG gives a more detailed insight and understanding in the relationship between hypoxia and glucose metabolism in NSCLC prior to and after chemotherapy. The hypoxic/metabolic distribution changes in a tumor are very variable in time and tumor location. This observation may have practical implications. It may allow selection for individualized treatment. An example is specific hypoxic radiotherapy in stage III disease or selection for pre-sensitization with hypoxia modifying agents during chemotherapy. Selecting patients for pre-sensitization may be important. Previously, no benefit could be demonstrated in a phase 3 study in unselected NSCLC of the addition of a hypoxia modifying agent (nitroglycerine) to a bevacizumab containing chemotherapy regimen [34]. On the other hand, a clear benefit was observed of adding tirapazamine (as an hypoxia modifying agent) to chemoradiotherapy in patients with head and neck cancer that were hypoxic according to ^18^F-FMISO PET [35]. But again, in a phase III trial with unselected patients, tirapazamine did not improve outcome [36].

### Limitations of this study

In this study protocol, we used 3 min per bed position in order to perform whole body imaging. This resulted in adequate images. After including the first patients, we started performing 15 min per bed position as well and only performed thoracic imaging. Using 15 min per bed position reduced signal noise and resulted in improved images. Because we chose to perform whole body scans, a 4D mode to compensate for breathing motion could not be used. However, the extent of breathing motion in our study was minimal; as in 5/7 patients, the primary tumor was located in the upper and middle lobe, respectively, and one patient had a metastasis of the clavicle only.

Although ^18^F-FMISO is studied most often, we used ^18^F-FAZA. ^18^F-FAZA shows superior biokinetics compared to ^18^F-FMISO [6, 10]. In addition, ^18^F-FMISO is more lipophilic and is cleared primarily via the hepatobiliary tract, while ^18^F-FAZA is mainly excreted renal, thereby giving less background noise to the scans [37]. However, as equally the case with all other hypoxic PET tracers, the ^18^F-FAZA images are more difficult to interpret then ^18^F-FDG due to the lower contrast with normal tissue.

Volumetric tumor analysis, although promising, has some limitations. Most importantly, the accuracy is dependent on good co-registration with the CT and on minimal tumor size changes. The use of this method will introduce variability within the measured values. At present, it is not clear whether this variability introduces clinically significant errors within the measurement. Nor is it known whether further fine-tuning of the software settings will limit the variability.

A concern inherent to the use of PET to study hypoxia is the resolution. The voxel size can be larger than the hypoxic area (especially when there is a combination of severe hypoxia and necrosis), and subsequently, such small hypoxic hotspots can potentially be classified as normoxic if the average value of the particular voxel is below the hypoxic cutoff value, and so leading to a false negative result [38].

## Conclusions

Different metabolic features of NSCLC are measured by ^18^F-FDG and ^18^F-FAZA. As measured with ^18^F-FAZA and ^18^F-FDG, tumor hypoxia is a dynamic phenomenon not associated with tumor metabolism, either before or after treatment in NSCLC patients with chemotherapy.
